# Research progress and applications of epigenetic biomarkers in cancer

**DOI:** 10.3389/fphar.2024.1308309

**Published:** 2024-04-12

**Authors:** Jianjun Gao, Wujiang Shi, Jiangang Wang, Canghai Guan, Qingfu Dong, Jialin Sheng, Xinlei Zou, Zhaoqiang Xu, Yifei Ge, Chengru Yang, Jiehan Li, Haolin Bao, Xiangyu Zhong, Yunfu Cui

**Affiliations:** ^1^ Department of Hepatopancreatobiliary Surgery, The Second Affiliated Hospital of Harbin Medical University, Harbin, China; ^2^ Department of General Surgery, Tangdu Hospital, Air Force Medical University, Xi’an, China

**Keywords:** epigenetics, cancer, DNA methylation, histone modification, non-coding RNA

## Abstract

Epigenetic changes are heritable changes in gene expression without changes in the nucleotide sequence of genes. Epigenetic changes play an important role in the development of cancer and in the process of malignancy metastasis. Previous studies have shown that abnormal epigenetic changes can be used as biomarkers for disease status and disease prediction. The reversibility and controllability of epigenetic modification changes also provide new strategies for early disease prevention and treatment. In addition, corresponding drug development has also reached the clinical stage. In this paper, we will discuss the recent progress and application status of tumor epigenetic biomarkers from three perspectives: DNA methylation, non-coding RNA, and histone modification, in order to provide new opportunities for additional tumor research and applications.

## 1 Introduction

**FIGURE 1:**
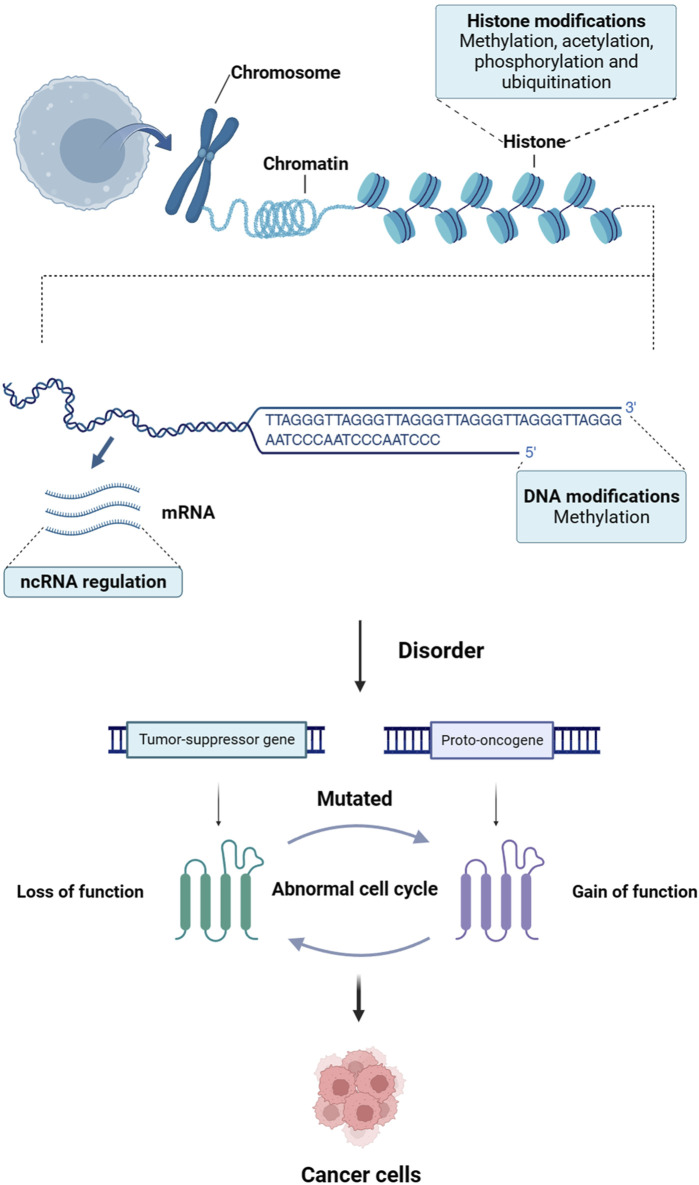
The link between epigenetics and cancer. Epigenetic disorders can lead to inactivation of tumor-suppressor genes and activation of proto-oncogenes, which play a crucial role in the initiation, progression, invasion and spread of cancer.

Cancer is a disease characterized by the disruption of critical pathways that control cellular processes such as repairing DNA, ensuring cell survival, promoting cell proliferation, and inducing cell death (Halbrook et al., 2023). Mutations in certain genes, including tumor suppressor genes and proto-oncogenes, contribute to the development of cancer (Riaud et al., 2024; Tuval et al., 2024). However, recent research has indicated that epigenetic abnormalities can also lead to the inactivation of tumor suppressor genes and activation of proto-oncogenes, playing a crucial role in the initiation, progression, invasion, and spread of cancer (Feinberg and Levchenko, 2023) (Figure 1). Epigenetics refers to a phenomenon in which gene function produces inheritable variations without altering the DNA sequence, ultimately resulting in changes in an organism’s phenotype (Lee and Kim, 2022). Epigenetic processes mainly encompass three categories: DNA methylation, modification of histones, and regulation of non-coding RNA (ncRNA) (Fitz-James and Cavalli, 2022).

Advances in high-throughput sequencing techniques have enhanced our comprehension of epigenetic mechanisms in cancer development and revealed numerous cancer-specific epigenetic biomarkers that offer potential as effective indicators for assessing tumor high-risk, facilitating early diagnosis, informing treatment decisions, and predicting prognosis (Lauschke et al., 2019). Currently, several pivotal regulatory molecules involved in epigenetics have been integrated into clinical tumor diagnosis for metastasis, recurrence, and prognostic assessment, serving as targets for clinical oncology therapies (Kravitz et al., 2023). This paper aims to discuss recent advancements and applications of tumor epigenetic biomarkers from three perspectives: DNA methylation, ncRNA, and histone modification. Consequently, it presents novel opportunities for further refinement of tumor therapies.

## 2 DNA methylation

DNA methylation is a biochemical process that involves the addition of a methyl group to a DNA base (Galbraith and Snuderl, 2022). This methyl group is commonly added to the 5′position of CpG island cytosine. This process is catalyzed by enzymes called DNA methyltransferases (DNMTs), which utilize S-adenosylmethionine as a methyl donor (Deng et al., 2023). In mammalian cells, DNMTs can be classified into three main types: DNMT1, DNMT3a, and DNMT3b. DNMT1 is responsible for maintaining the existing DNA methylation state, as well as catalyzing hemimethylated DNA and participating in DNA methylation repair (Yan et al., 2023). On the other hand, DNMT3a and DNMT3b catalyze the addition of new methyl groups to unmethylated DNA, converting it into methylated DNA (Lyko, 2018). DNA methylation plays a crucial role in regulating various biological processes within the mammalian genome. These processes include transcription and post-transcriptional processing, post-translational modification, chromatin remodeling, genomic imprinting, X chromosome inactivation, and inhibition of repetitive DNA elements (Saghafinia et al., 2018).

During tumorigenesis, there is typically a decrease in the overall level of intracellular DNA methylation. However, there is abnormal elevation of DNA methylation in the CpG island regions of specific genes (Ghosh et al., 2022). This abnormal methylation pattern leads to genome-wide instability and altered gene expression profiles (Liang and Weisenberger, 2017). Interestingly, it has been observed that proto-oncogenes tend to have reduced DNA methylation levels during tumorigenesis. This reduction in methylation promotes the elevated expression of these proto-oncogenes, potentially contributing to tumor development (Casalino and Verde, 2020). Conversely, tumor suppressor genes often exhibit increased DNA methylation levels, which inhibits their expression and further promotes tumor growth. Recent studies have also shown that specific methylation patterns can be detected in circulating tumor cells, blood, urine, and other bodily fluids (Palanca-Ballester et al., 2021). These methylation patterns, known as tumor-specific methylation genes, have become valuable tools for early tumor diagnosis and prognosis (Oliver et al., 2022). For example, many studies have discussed DNA methylation as a potential diagnostic biomarker in early-stage ovarian cancer (Singh et al., 2019). Singh et al. (Singh et al., 2020) revealed that HOXA9 and HIC1 can be used as suitable diagnostic serum biomarkers for early screening of ovarian cancer. Moreover, researchers have utilized a phenomenon called CpG Island methylator phenotype (CIMP) to characterize different subtypes of gastric cancer (Tahara et al., 2019). In the field of breast cancer research, DNA methylation features have also proven useful in identifying different subtypes of breast cancer (Ma et al., 2023).

### 2.1 Common tumor biomarkers of DNA methylation

#### 2.1.1 CIMP

CIMP is a CPG island that is abundant at CPG sites in the promoter region of DNA and exhibits a greatly methylated form (Suzuki et al., 2014). It can catalyze the transfer of DNMT from S-adenosine methionine to cause conformational changes in certain regions of DNA that affect protein-DNA interactions (Teodoridis et al., 2008). When methylation reaches a certain level, the regional DNA structure shrinks, the helix deepens, and the primordial regions on which numerous protein factors rely for binding shrink into large grooves, which is not conducive to transcription initiation (Sharma et al., 2010). It can cause the silencing of transcriptional genes or the inactivation of DNA repair genes and tumor suppressor genes, leading to tumor development. In 2006, Weisenberger’s research group in the United States recommended a simple and sensitive CIMP marker gene (CACNA1G, IGF2, NEUROG1, RUNX3 and SOCS1) to distinguish CIMP-positive colorectal cancer (Weisenberger et al., 2006). ​Moreover, CIMP is strongly associated with various molecular phenotypes such as elevated microsatellite instability, Tp53 wild-type, KRAS wild-type, BRAF variant, PIK3CA variant, and MLH1 methylation (Advani et al., 2018). The present study combines CIMP with additional tumor molecular biomarkers such as BRAF and KRAS for colorectal cancer diagnosis and prognosis prediction.

#### 2.1.2 CDO1

The CDO1 gene, also known as cysteine dioxygenase 1, is a metalloenzyme that contains iron and lacks a heme structure (Chen M. et al., 2023). Its main function is to convert cysteine into cysteine sulfite, which is crucial for the synthesis of taurine (Chen M. et al., 2023). Cysteine metabolism plays a key role in cell drying by regulating reactive oxygen species, especially CDO1, which plays a crucial role in inducing apoptosis by significantly increasing the production of ROS (Yamashita et al., 2018). Moreover, the CDO1 protein can interact with peroxisome proliferation-activating receptor (PPAR) γ, leading to the activation of CCAAT-enhancer-binding proteins (CEBP) α, an essential onco-transcriptional factor (Deng et al., 2015; Zhao et al., 2016). Hence, it is possible that CDO1 has a significant impact on inhibiting tumor growth during tumorigenesis. Numerous recent studies have identified elevated frequencies of DNA methylation in the CDO1 gene across various cancer types (Yamashita et al., 2018). Methylation of the CDO1 gene can be readily detected in the plasma and urine of lung cancer patients. When combined with other highly correlated methylated genes, it exhibits a sensitivity of 73% and specificity of 92% for diagnosing lung cancer (Liu et al., 2020b). Furthermore, CDO1 hypermethylation in various body fluids, apart from plasma, may aid in preoperative disease diagnosis. For instance, cervical scratches are a crucial source of evidence for the molecular detection of endometrial cancer. CDO1 hypermethylation in cervical scratches demonstrates a sensitivity of 85% and specificity of 88% for detecting endometrial cancer (Liew et al., 2019). CDO1 hypermethylation detection can also be beneficial in intraoperative diagnosis during gastric cancer surgery (Harada et al., 2019) and in evaluating routine biopsy samples for determining tumor eradication after neoadjuvant therapy for esophageal cancer (Ushiku et al., 2017).

#### 2.1.3 Septin 9

In the realm of cellular division and control of the cell cycle, the gene Septin 9 plays a crucial role (Sun et al., 2020). The methylation of Septin 9 gene promoter within plasma was initially confirmed in the year 2008 (Lofton-Day et al., 2008). To assess the viability of Septin 9 as a marker in colorectal cancer screening, a comprehensive investigation known as the PRESEPT study was carried out. The outcomes revealed an overall sensitivity of 50% and a specificity of 91% for the detection purposes (Church et al., 2010). In a study comparing the plasma Epi proColon test to a fecal immunochemical test (FIT), it was observed that Septin 9 exhibited identical sensitivity (73%) to FIT, but its specificity (81.5% vs 97%) was considerably lower (Johnson et al., 2014).

The second-generation Septin 9 assay, termed Epi proColon 2, displayed enhanced specificity of 87% when contrasted with 82% in the control group (Jin et al., 2015). Additionally, Epi proColon 2 showcased a remarkable detection sensitivity of 75%, surpassing that of the FIT trial (75% vs 58%) (Wu et al., 2016). In a 2016 study conducted by Song et al. (Song et al., 2016), three distinct detection methods for Septin 9 were compared, ultimately revealing that Epi proColon demonstrated superior sensitivity but relatively lower specificity, with both figures hovering around 82%. On the other hand, the effectiveness of Epi proColon 2.0 mirrored that of the commercially available Seni-Colon assay, which has been approved by the China Food and Drug Administration. These assays demonstrated sensitivity ranging from 75% to 77% and specificity ranging from 96% to 97% (Loktionov, 2020).

#### 2.1.4 NDRG4 and BMP3

NDRG4, a member belonging to the N-myc downstream regulatory gene family, encodes a protein that is specifically expressed in brain and heart tissues (Zhou et al., 2001). The involvement of NDRG4 revolves around cell proliferation, apoptosis, and cell differentiation, and it has been linked to various types of cancer (Melotte et al., 2010). BMP3, which is a type of bone morphogenetic protein, was initially reported in 2005 as one of the tumor suppression factors in colorectal cancer (Koinuma et al., 2005). In a study conducted in 2012, where 252 patients with colorectal cancer and 293 subjects with negative results from colonoscopy were screened, Cologuard (KRAS mutation + fecal hemoglobin + NDRG4 and BMP3 methylation) exhibited a sensitivity of 85% and specificity of 90% (Ahlquist et al., 2012). Furthermore, a study involving 9,989 participants in 2014 demonstrated that the Cologuard test achieved a sensitivity of 92% for colorectal cancer, 42% for precancerous lesions, and a specificity of 90%. In comparison, the FIT test had lower sensitivity (74%) but higher specificity (96%) (Imperiale et al., 2014). At present, a multicenter prospective clinical trial based on Cologuard 2.0 is underway, and the results are currently pending (NCT04144751).

#### 2.1.5 MGMT

MGMT is a greatly efficient DNA methyltransferase that transfers methyl groups from DNA molecules to their own amino acid residues (Fang, 2024). MGMT can repair damage to cellular DNA caused by alkylation of various agents and prevent DNA mutations caused by methylation, and thereby preventing cancer (Butler et al., 2020). Levels of MGMT expression in normal tissues are very low under physiological conditions, but increase under alkylating agents or radiation (Chen Y. et al., 2023). A 2005 randomized trial showed that patients with methylated MGMT promoters had a greater survival benefit than patients with unmethylated MGMT promoters in glioblastoma patients treated with combination radiotherapy and temozolomide chemotherapy (Hegi et al., 2005). Therefore, glioblastoma can be divided into two categories based on whether or not there is methylation of the MGMT promoter. Among them, the unmethylated patients were resistant to temozolomide and had poor efficacy (Hegi et al., 2008). The 2018 NCCN guidelines clearly suggest that MGMT is an important prognostic and outcome predictor of glioblastoma. In addition to predicting patient response to chemotherapy, MGMT methylation can also be used to predict radiotherapy response and prognosis for glioblastoma without adjuvant alkylating chemotherapy (Wick et al., 2012). At present, MGMT test kits have been developed and efforts are underway to find more sensitive and accurate means of detection.

#### 2.1.6 LINE-1

LINE-1, also known as long-spread repeat 1, is a type of non-LTR retrotransposon (Mendez-Dorantes and Burns, 2023). Its transposition has the potential to cause changes and rearrangements in the DNA of host cells, which can lead to the development of various serious genetic diseases such as cancer (Kerachian and Kerachian, 2019). In normal tissues, LINE-1 is heavily methylated, but in tumor tissues, its methylation status is reduced (demethylated) (Lavia et al., 2022). Research has demonstrated that demethylated LINE-1 can serve as a reliable biomarker for detecting lung cancer (Stading et al., 2021). P16 is an important tumor suppressor gene located on human chromosome 9p21 (Serra and Chetty, 2018). It functions by inhibiting the growth of tumor-killing cells (Muss et al., 2020). Several studies have indicated that hypermethylation of the p16 gene is strongly associated with poor prognosis in patients diagnosed with colorectal cancer (Karam et al., 2019), liver cancer (Wong et al., 1999), and lung cancer (Peng et al., 2023). Another gene involved in cancer development is glutathione S-transferase 1 (GSTP1). Research has shown that the absence of GSTP1 promoter methylation expression in prostate cancer is associated with a sensitivity of 73%, specificity of 100%, positive predictive value of 100%, and negative predictive value of 78% (Miyake et al., 2012).

### 2.2 Inhibitors of DNA methylation

One of the earliest epigenetic drugs used to treat cancer were DNMT inhibitors (DNMTis) (Zhou et al., 2018). Currently, DNMTis developed for abnormal DNA methylation are divided into nucleoside inhibitors and non-nucleoside inhibitors.

#### 2.2.1 Nucleoside inhibitors

​Nucleoside inhibitors primarily include azacitidine, decitabine, zebularine and derivatives of cytosine nucleosides as substrates. Azacitidine is the first epigenetic drug approved by the FDA for the treatment of cancer (Myasoedova et al., 2019). It is a nucleoside analogue of cytidine that can specifically trap DNMT and thus achieve the effect of inhibiting DNA methylation (Raslan and Garcia-Horton, 2022). Azacitidine is mainly used to treat myelodysplastic syndromes (MDS) (Merz et al., 2024). The other drug approved by the FDA is decitabine (Myasoedova et al., 2019). It works by binding to DNA, causing DNA methylation and stopping DNA replication during the S phase (Dhillon, 2020). In 2020, the FDA approved an oral fixed-dose combination tablet containing deccitabine and cedazuridine (DEC-C) for the treatment of MDS (Kim et al., 2022). Zebularine is a cytidine DNA methylation inhibitor containing 2-(1H) pyrimidine cycloketone, which can effectively remove the DNA methylation modification of p16 gene in the hypermethylation modification state by covalently binding with DNMT (Andrade et al., 2017). Currently, Zebularine is in preclinical experiments for the treatment of breast cancer and certain blood tumors (Marquez et al., 2005; Chen et al., 2012). The next-generation of DNMTis includes guadecitabine, which is a dinucleotide prodrug of decitabine (Daher-Reyes et al., 2019). It combines decitabine and guanosine into one molecule (Wong et al., 2022). The longer half-life and better bioavailability make guadecitabine well tolerated in myelodysplastic syndrome and acute myeloid leukaemia patients with MDS (Issa et al., 2015; Kantarjian et al., 2017; Sébert et al., 2019). A phase Ⅰ dose-escalation trial demonstrated that guadelitabine combined with pembrolizumab is tolerated and has biological and anticancer activity in patients with advanced solid tumors (Papadatos-Pastos et al., 2022). In addition, azacitidine derivative CP-4200 contains hydrophobic fragments that improve its delivery to cancer cells, making it independent of the activity of the nucleoside transporter mechanism (Hummel-Eisenbeiss et al., 2013).

#### 2.2.2 Non-nucleoside inhibitors

​Non-nucleoside inhibitors do not contain the cytosine nucleoside skeleton structure and can bind directly to methylated regions in DNMT, rendering the enzyme ineffective (Del Castillo Falconi et al., 2022). ​Procaine is a benzoic acid compound with local anesthetic and anti-arrhythmic activity, but it has been found to inhibit the growth and demethylate liver cancer cells by treating them (Tada et al., 2007). Similar to Procaine, Procainamide is also a derivative of aminobenzoic acid, which is currently used to treat conditions such as anxiety and irregular heartbeat, and also has demethylation effects (Lee et al., 2005). Gao et al. found that procaine and procainamine may have potential use in preventing the development of lung cancer (Gao et al., 2009). Hydralazine is commonly used to treat high blood pressure and heart failure (Chaemsaithong et al., 2024). The study showed that hydrazine was able to restore the expression of tumor suppressor genes in cancer cell lines and primary tumors where promoter hypermethylation was silenced (Segura-Pacheco et al., 2003). In a phase II, single-arm study, hydralazine and valproate increased the chemotherapy efficacy of doxorubicin and cyclophosphamide (Arce et al., 2006). Then, a phase II study found that hydralazine and valproate could overcome chemotherapy resistance in patients with advanced refractory solid tumors (Candelaria et al., 2007). A randomized phase III demonstrates that epigenetic therapy (hydralazine and valproate) has a significant advantage over the current standard combination chemotherapy in terms of progression-free survival of cervical cancer (Coronel et al., 2011). In addition, a randomized, double-blind phase III trial comparing hydrazine valproate with placebo is ongoing (Saito et al., 2010). Epigallocatechin-3-gallate (EGCG) are bioactive polyphenol compounds that regulate epigenetic changes, including DNA methylation and histone modification (Włodarczyk et al., 2024). EGCG has become an important pathway for epigenetic therapy for leukemia and MDS, and it also has synergies when used in combination with conventional chemotherapy drugs (Della Via et al., 2023). In addition, in phase Ⅱ double-blind, placebo-controlled randomized clinical trial, preventive use of EGCG solution significantly reduced the incidence and severity of RID in patients with adjuvant radiotherapy for breast cancer (Zhao et al., 2022). And, in a randomized, double-blind trial, EGCG showed good tolerance, but across the study population as a whole, there was no statistically significant difference in adenoma rates between the EGCG and placebo groups (Seufferlein et al., 2022). Countless different anti-tumor applications of these drugs are still in the research stage, such as SGI-1027, RG108 analogues, Quinazoline, Propiophenone, and Pyrrolopyridine derivatives (Pechalrieu et al., 2017) (Table 1).

**TABLE 1:** Summary of commonly DNMTis.

	Drugs	Status	Type of cancer targeted	Clinical Trials.gov Identifier
Nucleoside DNMTis	Azacitidine	FDA-approved	AML, MDS, Head and Neck Cancer, Pancreas Cancer	NCT03019003 NCT03264404
	Decitabine	FDA-approved	AML, MDS, CMML, Metastatic Renal Cell Carcinoma, Primary Malignant Neoplasm of Ovary, Prostate Carcinoma, Breast Cancer	NCT04049344 NCT02159820 NCT03709550 NCT02957968 NCT03295552
	Guadecitabine	In clinical trial	Colorectal Adenocarcinoma, Advanced Kidney Cancer, Small Cell Lung Cancer	NCT03576963 NCT03308396 NCT03913455
Non-nucleoside DNMTis	Hydralazine	In clinical trial	Advanced cervical cancer, Locally Advanced breast cancer, Ovarian Cancer, Rectal Cancer, Lung Cancer	NCT00533299 NCT00532818 NCT00575640 NCT00395655 NCT00996060
	EGCG	In clinical trial	Breast Cancer, Colon Cancer, Urothelial Carcinoma, Lung Cancer, Triple Negative Breast Cancer, Bladder Cancer, Prostate Cancer	NCT02580279 NCT02891538 NCT01993966 NCT02577393 NCT05680662 NCT00666562 NCT00676780

## 3 Histone modification

Histone proteins are the basic structural proteins of eukaryotic chromosomes and are a class of minor molecular alkaline proteins, with 5 types: H1, H2A, H2B, H3 and H4 (Fang et al., 2021). The histone amino terminal (n-terminal) domain extends out of the nucleosome and can interact with additional regulatory proteins and DNA (Robert and Jeronimo, 2023). Histone modifications include methylation, acetylation, ubiquitination, crotonylation and phosphorylation (Zaib et al., 2022). It can be passed from generation to generation in cells as a marker, thus constituting a “histone code” to effectively regulate specific genes. ​Imbalanced histone modification can lead to tumor development, and methylation and acetylation loss of histone H3 and H4 residues have been shown to be markers of tumor cells (Ray-Gallet and Almouzni, 2022). Histone modification is expected to be an effective epigenetic tumor marker.

### 3.1 Common tumor biomarkers of histone modification

#### 3.1.1 Histone methylation modification

Histone methylation, which occurs on the amino terminal arginine or lysine residues of H3 and H4 histones, is a significant biochemical process (Huang et al., 2024). This methylation is mainly facilitated by histone methyltransferase (HMT), which can be further categorized into histone lysine methyltransferase (HKMT) and protein arginine methyltransferase (PRMT) (Patnaik et al., 2023). On the other hand, histone demethylases can be broadly classified into the lysine-specific demethylase (LSD) family and the JmjC domain-containing family (JMJD) (Tong et al., 2024). Methylation can occur at various sites, including H3 lysine (H3K) sites 4, 9, 27, 36, 79, and 20 of H4 lysine (H4K) (Hyun et al., 2017). ​According to several studies, specific methylation patterns, such as H3K9me3, H3K27me3, H3K36me3, and H4K20me3, serve as vital indicators for gastric, liver, breast, pancreatic, ovarian, and colon cancers (Sasidharan Nair et al., 2018; Sogutlu et al., 2022; An et al., 2023; Burlibasa et al., 2023; Tachaveeraphong and Phattarataratip, 2024). ​ Furthermore, the expression of H3K9me2, H3K9me3, and H3K27me3 is significantly associated with clinical pathologies and may serve as independent risk factors for survival assessment in patients with gastric cancer (Li et al., 2019).

#### 3.1.2 Histone acetylation modification

Histone acetylation is a reversible homeostatic process. The dynamical balance between histone acetylation and deacetylation is an influential factor in maintaining the stability of gene expression and chromatin structure, and disruption of this dynamical balance can lead to abnormal gene expression and lead to the occurrence and development of tumors (Zhao S. et al., 2018). This homeostasis is essentially co-regulated by histone acetyltransferase (HAT) and histone deacetylase (HDAC) (Audia and Campbell, 2016). HAT can add acetyl groups to lysine to make it positively charged, causing chromatin structures to open and promoting gene transcription, while HDAC can remove acetyl groups from lysine, reversing this process and inhibiting transcription (Dang and Wei, 2022). Studies have suggested that H3K9ac and H3K27ac may be markers of hepatocellular carcinoma and pancreatic cancer (Liu Y.-X. et al., 2020; Ono et al., 2021). Dou et al. (Dou et al., 2023) explained that H3K27 acetylation upregulates LINC00501 to promote gastric cancer metastasis through activation of epithelial-mesenchymal transformation and angiogenesis.

#### 3.1.3 Histone phosphorylation modification

Histone phosphorylation is the phosphorylation of amino acid residues at the N terminal of a histone (Zhang et al., 2014). Its main types include histone H1 phosphorylation, histone H2A/H2B phosphorylation, histone H3 phosphorylation, and histone H4 phosphorylation, among others (Baker et al., 2010). The mechanism of histone phosphorylation may be due to the negative charge carried by the phosphate group neutralizing the positive charge on the histone, resulting in a decrease in the affinity between the histone and DNA (Rossetto et al., 2012). It is also possible to modify surfaces that can bind to protein recognition modules and interact with specific protein complexes. These two mechanisms affect the structure and function of chromosomes and are involved in physiological processes such as cell division (Ajiro et al., 2010). Currently, although histone phosphorylation has been extensively studied (Köhler et al., 2012; Abbaoui et al., 2017), its potential role as a tumor marker remains to be explored.

#### 3.1.4 Histone ubiquitination and crotonylation modification

The modification sites for histone ubiquitination are located primarily on the C-terminal lysine residues of H2A, H2B, H3, and connexin H1 (Mattiroli and Penengo, 2021). Ubiquitination can initiate the transcription of target genes through interactions with other histone modifications and as a recruitment signal for transcription factors (Mattiroli and Penengo, 2021). Studies have found that an increase in H2AK119Ub1 and a decrease in H3K27Me3 may be markers of molecular staging of pancreatic cancer (Chen et al., 2014).

The concept of Crotonylation was first proposed in 2011, which can occur on lysine (K) residues of both histone and non-histone proteins and has similarities with acetylation in its structure, recognition proteins and regulated enzyme systems (Tan et al., 2011; Sabari et al., 2015). Although little research has been done, it plays an important role in regulating transcriptional activity, stress protection from kidney injury, and spermatogenesis and development (Ruiz-Andres et al., 2016) (Table 2).

**TABLE 2:** Epigenetic biomarkers in cancers.

		Name	Cancer	References
DNA methylation		CDKN2A	Liver cancer	Zucman-Rossi et al. (2015), Moldogazieva et al. (2021)
		RUNX3		Krajnović et al. (2023)
		RASFF1A	Liver cancer, Lung cancer, Prostate cancer, Colorectal cancer, Melanoma, Oral cancer	Payne et al. (2009), Salvianti et al. (2015), Lim et al. (2016), Liu et al. (2020a), Raos et al. (2021)
		CDO1	Lung cancer, endometrial cancer	Liew et al. (2019), Liu et al. (2020b)
		SEPT9	Colorectal cancer	Johnson et al. (2014), Jin et al. (2015)
		CIMP		Kasprzak (2023)
		VIM		Müller and Győrffy (2022), Brenne et al. (2023)
		NDRG4		Ahlquist et al. (2012), Imperiale et al. (2014)
		BMP3		
		SDC2		Oh et al. (2013)
		RARB2		Barault et al. (2018)
		BCAT1, IKZF1		Bhangu et al. (2018)
		MGMT	Glioblastoma	Hegi et al. (2005), Hegi et al. (2008), Wick et al. (2012)
		CDH1	Gastric cancer	Hansford et al. (2015), Gamble et al. (2021)
		CDKN2A		Xu et al. (2021)
		CD1D	Pancreatic cancer	Kisiel et al. (2015), Majumder et al. (2020)
		BRCA1/2	Breast cancer	Tutt et al. (2018), Brahim et al. (2022)
		PITX2		Absmaier et al. (2018)
		RARB2	Breast cancer, lung cancer	Buhmeida et al. (2011), Palanca-Ballester et al. (2021)
		SHOX2	Lung cancer	Song et al. (2015), Wei et al. (2021)
		PTGER4		Weiss et al. (2017)
		PCDHGA12		Jeong et al. (2018)
		LINE-1	Colorectal cancer, Liver cancer, Lung cancer	Wong et al. (1999), Karam et al. (2019), Peng et al. (2023)
		GSTP1	Prostate cancer, Breast cancer	Zhao et al. (2018a)
		TIMP3	Oral cancer	Lim et al. (2016)
Histone modification	Methylation	H3K9me3	Gastric cancer, liver cancer	Ji et al. (2019), Li et al. (2019)
		H3K27me3	Ovarian cancer, breast cancer, pancreatic cancer, gastric cancer	Hu et al. (2021), Chen et al. (2022), Day et al. (2022), Marsolier et al. (2022)
		H3K36me3	Liver cancer	Nepal and Andersen (2023)
		H4K20me3	Astrocytome, breast cancer	Leszinski et al. (2012), Klonou et al. (2021), Wang et al. (2023)
	Acetylation	H3K9ac	Oral cancer	Sant'Ana et al. (2020)
		H3K27ac	Gastric cancer, liver cancer	Zhao et al. (2020), Ho et al. (2023)
	Ubiquitination	H2AK119Ub	Pancreatic cancer	Yan et al. (2021)
ncRNA	miRNA	miR-10b	Pancreatic cancer	Preis et al. (2011)
		miR-16	Lung cancer	Wang et al. (2013)
		miR-34a		Gallardo et al. (2009)
		miR-21	Breast cancer, colorectal cancer, lung cancer, prostate cancer	Yan et al. (2008), Saito et al. (2011), Yaman Agaoglu et al. (2011), Toiyama et al. (2013)
		miR-221	Prostate cancer	Yaman Agaoglu et al. (2011)
		miR-375		Huang et al. (2015)
		miR-506	Ovarian cancer, pancreatic cancer, gastric cancer	Yang et al. (2013), Sakimura et al. (2015), Li et al. (2016)
		miR-1290	Colorectal cancer, prostate cancer	Huang et al. (2015), Imaoka et al. (2016)
	lncRNA	CamK-A	Breast cancer	Sang et al. (2018)
		EPIC1		Wang et al. (2018)
		LINK-A		Lin et al. (2016)
		CCAT1	Colorectal cancer	McCleland et al. (2016)
		CCAT2		Ozawa et al. (2017)
		FAL1	Ovarian cancer	Hu et al. (2014)
		H19	Gastric cancer	Zhou et al. (2015)
		HOTAIR	Ovarian cancer, breast cancer, pancreatic cancer, colorectal cancer, ESCC	Gupta et al. (2010), Kogo et al. (2011), Kim et al. (2013), Li et al. (2013), Teschendorff et al. (2015)
		HOTTIP	Liver cancer	Quagliata et al. (2014)
		HULC		Panzitt et al. (2007)
		lncARSR	RCC	Qu et al. (2016)
		MALAT1	Lung cancer, prostate cancer	Ji et al. (2003), Ren et al. (2013)
		NEAT1	Prostate cancer	Chakravarty et al. (2014)
		PCA3		Hessels et al. (2003)
		PCAT-1		Prensner et al. (2011)
		PCAT-14		Shukla et al. (2016)
		SChLAP1		Prensner et al. (2014)
		UCA1	Bladder cancer	Wang et al. (2006)
	circRNA	circAR	Prostate cancer	Vo et al. (2019)
		circCCDC66	Colorectal cancer	Hsiao et al. (2017)
		ciRS-7		Weng et al. (2017)
		circCTNNB1	Gastric cancer	Yang et al. (2019a)

circRNA, circular RNA; ESCC, esophageal squamous cell carcinoma; lncRNA, long non-coding RNA; RCC, renal cell carcinoma; miRNA, microRNA.

### 3.2 Inhibitors of histone modification

**FIGURE 2:**
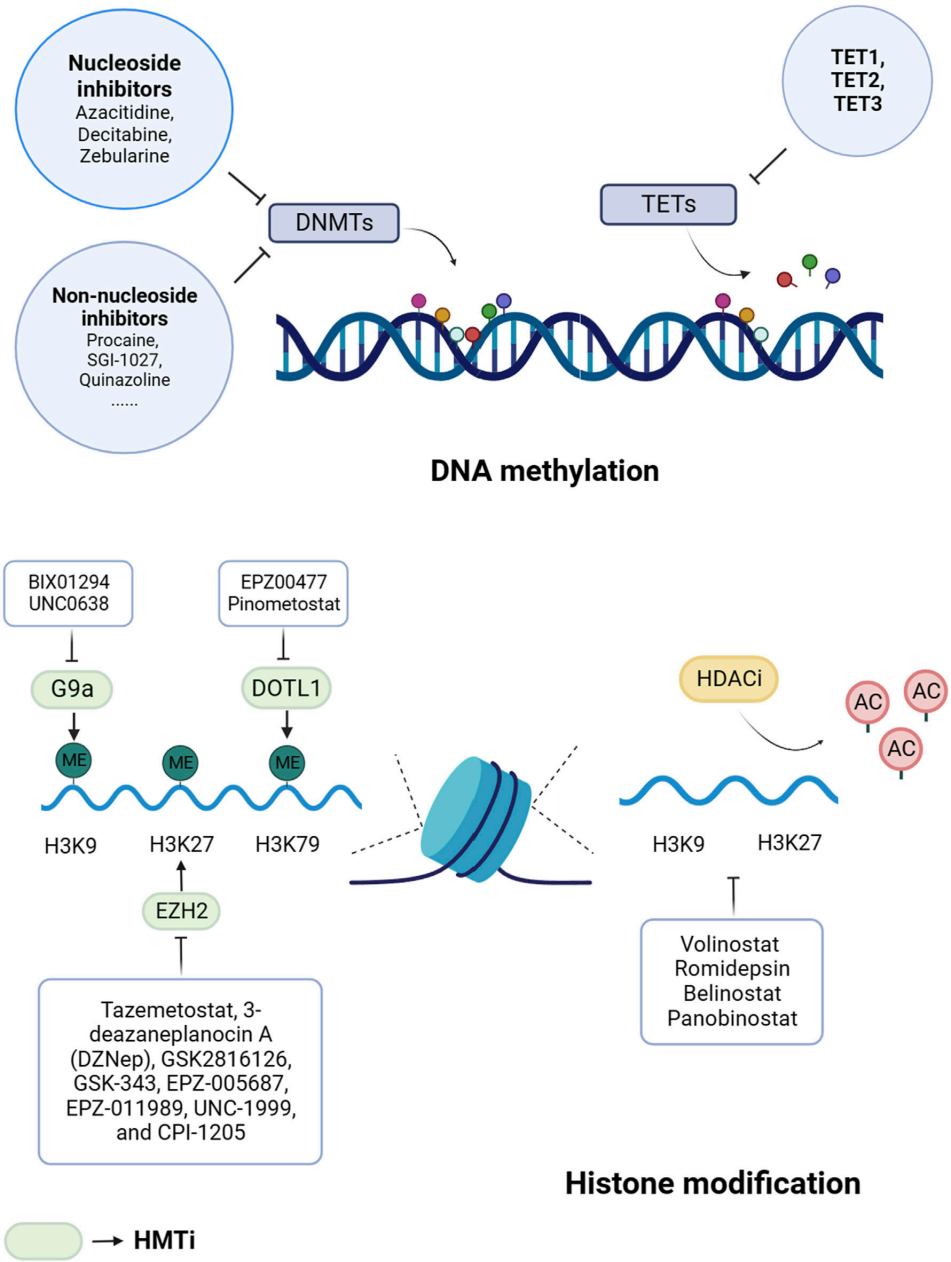
Epigenetic drugs for cancer treatment. Histone methyltransferases inhibitors (HMTis) and histone deacetylases inhibitors (HDACis) are the main histone methyltransferases inhibitors. DNMTs, DNA methyltransferases; TETs, ten-eleven translocation enzymes; H3, histone 3; H4, histone 4; K, lysine; ME, methylation; AC, acetylation; DOT1L, DOT1-like histone lysine methyltransferase; EZH2, enhancer of zeste homolog 2.

Currently, histone methyltransferases inhibitors (HMTis) and histone deacetylases inhibitors (HDACis) are the main histone methyltransferases inhibitors in clinical trials (Epp et al., 2023). There are relatively few studies of additional types of histone-targeting inhibitors (Figure 2).

#### 3.2.1 HMTi

##### 3.2.1.1 EZH2

EZH2 is an important enzyme involved in the activity of the PRC2, which is responsible for the methylation of histone H3K27 (Guo et al., 2024). This methylation process helps in the formation of heterochromatin and the inhibition of transcription (Lee et al., 2022). EZH2 has the ability to promote the growth and spread of various types of tumors, including melanoma (White et al., 2021), oral squamous cell carcinoma (Zhou et al., 2023), and breast cancer (Velez et al., 2024). There are several EZH2 inhibitors, including Tazemetostat, 3-deazaneplanocin A (DZNep), GSK-343, EPZ-005687, EPZ-011989, UNC-1999, and CPI-1205, that have shown promising results in preclinical studies (Wan et al., 2023). However, their efficacy and safety need to be further evaluated in clinical trials. Currently, Tazemetostat is undergoing phase Ⅰ/II clinical trials for the treatment of various tumors (Italiano et al., 2018; Gounder et al., 2020; Morschhauser et al., 2020; Sarkozy et al., 2020; Zauderer et al., 2022). Additionally, CPI-1205 has shown strong antitumor effects in xenograft models and is undergoing phase I clinical trials (Vaswani et al., 2016). In a phase I study, GSK2816126 demonstrates moderate anticancer activity at tolerated doses in solid tumors or B-cell lymphomas (Yap et al., 2019).

##### 3.2.1.2 DOT1L

DOT1L is responsible for the methylation modification of histone H3K79 and is also an important target for drug selection of methylase inhibitors (Alexandrova et al., 2022). Yang et al. (Yang L. et al., 2019) found that the use of DOT1L inhibitor EPZ004777 could induce cell cycle arrest of colorectal cancer cells. Another DOT1L inhibitor, EPZ-5676, showed promising results in a phase I clinical trial in MLL-rearranged leukemia (Stein et al., 2018) and was validated as ‘orphan drug’ toward MLL-rearranged leukemia by FDA (Cao et al., 2021).

##### 3.2.1.3 G9a

G9a (also known as euchromatic histone-lysine N-methyltransferase 2, EHMT2) and G9A-like protein (GLP) are lysine methyltransferases that catalyze H3K9me1 and H3K9me2 and methylation modification of H3K27 (Haebe et al., 2021). G9a and GLP form a complex in the body that has been shown to promote tumor cell growth and affect cell cycle and metabolic pathways (Shinkai and Tachibana, 2011). Studies have found that G9a is overexpressed in esophageal squamous cell carcinoma, hepatocellular carcinoma, invasive lung cancer, brain cancer, multiple myeloma, and invasive ovarian cancer (Casciello et al., 2015). BIX01294 is the first substrate-competitive inhibitor that selectively inhibits G9a/GLP and downregulates H3K9me2 expression. However, BIX01294 has weak activity in cell assays and is cytotoxic at concentrations higher than 4.1 μmol/L (Kaniskan and Jin, 2015). UNC0638 is a further synthetic compound based on UNC0224 and UNC0321 that significantly downregulates H3K9me2 in a variety of cells. However, UNC0638 has poor pharmacokinetics *in vivo* and is not suitable for animal studies (Kaniskan et al., 2018).

#### 3.2.2 HDACis

The HDACis have shown potential in inhibiting the growth of cancer cells, as well as inducing cancer cell differentiation and apoptosis by suppressing HDAC activity and promoting histone acetylation (Ramaiah et al., 2021). These inhibitors can be classified into four categories based on their chemical structure: short-chain fatty acids, hydroxamic acids, cyclic peptides, and benzamide derivatives (Shanmugam et al., 2022). In the United States, the FDA has currently approved four commonly used HDACis for the treatment of various hematologic tumors and solid tumors (Zhang et al., 2015).

##### 3.2.2.1 Volinostat

Volinostat, also called nitrosamine hydroxamic acid (SAHA), is a drug that belongs to the hydroxamic acid category. Its approval by the US FDA in 2006 for treating cutaneous T-cell lymphoma (Siegel et al., 2009) marked a significant milestone. In the context of solid tumors, Volinostat is capable of inducing apoptosis in prostate tumor cells by blocking the Akt/FOXO3a signaling pathway (Shi et al., 2017). This inhibition can prevent the development of castration-resistant prostate cancer, which typically arises due to conventional therapies such as androgen deprivation therapy. Additionally, Volinostat reduces the adverse effects associated with drug resistance and dose toxicity induced by chemotherapy drugs such as Paclitaxel (Shi et al., 2017). Moreover, SAHA exhibits potential in improving the radio sensitivity of pancreatic cancer cells, making it a promising agent for enhancing the efficacy of radiotherapy against pancreatic cancer in the future (Wu et al., 2017). Notably, a clinical trial (NCT00731731) investigated the combination of Volinostat, Temozolomide, and radiotherapy for early treatment of glioblastoma multiforme has been reported. The positive anti-tumor effects of SAHA notwithstanding, it is crucial to highlight the elevated toxicity observed with high doses of the drug, resulting in adverse effects like fatigue, diarrhea, anorexia, dehydration, myelosuppression, thrombocytopenia, among others (Mrakovcic et al., 2017). ​ As a consequence, enhancing the selectivity of HDACis or refining existing HDACis based on SAHA’s essential pharmacophore group holds great potential for future applications.

##### 3.2.2.2 Romidepsin

FK228, also referred to as Romidepsin, is a member of the cyclic peptide class of inhibitors. In 2012, the approval for the treatment of cutaneous T cell lymphomas (CTCL) and peripheral T-Cell lymphomas (PTCL) was granted by the US FDA (Mottamal et al., 2015). HDAC1 and HDAC2 are directly inhibited by Romidepsin (Saijo et al., 2015). In cell culture experiments, it exhibits similar effects to SAHA, as it has the capability to impede cancer cell growth and induce both DNA double-strand break damage and cell apoptosis simultaneously (Pojani and Barlocco, 2021). Additionally, other research has demonstrated the effectiveness of combining Romidepsin with chemotherapy drugs for the treatment of non-small cell lung cancer (NSCLC) (Karthik et al., 2014).

##### 3.2.2.3 Belinostat

Belinostat, an HDACis medication approved by the FDA in 2014 specifically for treating PTCL. It is a hydroxylate that effectively inhibits the activity of both class I and class II HDACs (McDermott and Jimeno, 2014). This drug carries significant anti-tumor properties, particularly against solid tumors. For instance, Belinostat has demonstrated its ability to induce apoptosis in PC3 cells, a human prostate cancer cell line, through a mitochondrial pathway during prostate cancer treatment (Molife and de Bono, 2011). This process effectively hinders the development and manifestation of prostate cancer (Gravina et al., 2012). Moreover, Belinostat disrupts the expression of ubiquitin-related proteins by triggering proteasomal degradation of the SOS protein and down-regulating downstream MAPK signal transduction. This interference effectively impacts the functioning of critical survival signals in lung squamous cell carcinoma, thereby promoting cellular apoptosis (Kong et al., 2017).

##### 3.2.2.4 Panobinostat

In 2015, the FDA approved Panobinostat for clinical trials as a treatment for multiple myeloma (Laubach et al., 2015). As a hydroxylated derivative, Panobinostat exhibits inhibitory properties on HDACs from class Ⅰ, class Ⅱ, and class IV (Biersack et al., 2022). Currently, Panobinostat is being utilized for the treatment of other tumor types, including thyroid cancer (NCT01013597), colorectal cancer, and prostate cancer (NCT00663832) (Losson et al., 2016). In a study conducted by Ferrari et al. (Ferrari et al., 2019), the efficacy and safety of combining Panobinostat with Bicalutamide for hormone-resistant prostate cancer were evaluated. The results demonstrated that this combination therapy could effectively prolong the progression-free survival time of hormone-resistant prostate cancer, while remaining tolerable in terms of its toxic effects.

Moreover, anti-tumor research also emphasizes the significance of SIRTs (sirtuins), which belong to the family of NAD (+)-dependent class III histone deacetylases. Although the FDA has not yet approved any drugs targeting SIRTs, numerous studies have highlighted the anti-tumor effects of SIRT inhibitors. One such inhibitor is Suramin, which selectively targets SIRT1 and SIRT2. Currently, clinical trials are underway to explore its potential use in various cancers (Villalba and Alcaín, 2012).

## 4 ncRNA modification

ncRNA refers to RNA that does not code for proteins, including microRNA (miRNA), long non-coding RNA (lncRNA) and circular RNA (circRNA), etc., they have important functions in life activities (Yan and Bu, 2021). miRNAs are small RNAs with a total length of about 22 nucleotides (nt). The miRNAs bind to specific target miRNAs via complementary sequences, thereby inducing miRNA division, degradation, or translation blocking (Vos et al., 2019). ​Both lncRNAs and circRNAs are longer than 200nt, but lncRNAs are linear while circRNAs are circular (Wu et al., 2023). LncRNAs and circRNAs regulate gene expression through multiple mechanisms. They can act as decoys for miRNAs, preventing the degradation of the targeted mRNA (Batista and Chang, 2013). They can regulate the binding of transcription factors to promoters and thus regulate the expression of target genes (Fatica and Bozzoni, 2014). They can also serve as scaffolds to regulate protein-protein interactions and associated downstream signaling pathways (Flynn and Chang, 2014).

### 4.1 Common tumor biomarkers of ncRNA

Research has demonstrated the existence of diverse categories of ncRNAs, such as miRNA, lncRNA, and circRNA, which exhibit differential expression patterns in malignant tissues compared to adjacent tissues (Wang et al., 2019; Yan and Bu, 2021). Consequently, ncRNAs hold great potential as diagnostic and prognostic biomarkers for tumors. Up to now, urinary PCA3 represents the sole FDA-approved molecular tumor diagnostic assay derived from ncRNA (PRO-GENSA PCA3) (Borbiev et al., 2023). Investigations have revealed that PCA3 serves as a specific indicator that is upregulated in prostate cancer and can be identified in patient urine (Chen J.-Y. et al., 2023).

Numerous ongoing clinical trials are currently examining the feasibility of utilizing ncRNAs as indicators for tumors (Zhou et al., 2022). A diagnostic study, conducted in a prospective, longitudinal, blinded, observational manner, involved 400 patients who underwent low-dose computed tomography (LDCT) screening specifically for lung cancer. The main objective of this study was to assess if the miRNA profile (HMBDx, exclusively licensed to Hummingbird) outperforms LDCT in diagnosing lung cancer (NCT03452514). Previous investigations have indicated that miRNA-10b exhibits higher expression levels in glioblastomas compared to normal brain tissue (El Fatimy et al., 2017). A clinical trial, comprising of around 200 patients diagnosed with glioblastomas, aimed to evaluate if the expression levels of miR-10b in primary tumor, blood, and cerebrospinal fluid samples could serve as prognostic and diagnostic markers for glioblastomas (NCT01849952). Moreover, a case-control observational study utilized next-generation sequencing to analyze the differential expression of miRNAs and lncRNAs in blood samples obtained from 160 patients diagnosed with high-grade serous ovarian cancer and benign gynecologic disease (NCT03738319).

### 4.2 Therapeutic targeting of ncRNAs in cancer

In the field of tumorigenesis and development, ncRNAs play a crucial role as regulatory molecules. Hence, there has been a significant focus on the development of efficient therapeutic techniques for suppressing (proto-oncogenes) or increasing expression (tumor suppressor genes) of ncRNAs (Rupaimoole and Slack, 2017; Arun et al., 2018). ​ Although most studies investigating ncRNA-targeted drugs for cancer are in the preclinical stage, they demonstrate the immense potential of this approach. For illustration, using antisense oligonucleotides, the deletion of lncRNA MALAT1 in animal models effectively impacts the growth and metastasis of lung and breast cancer cells (Gutschner et al., 2013; Arun et al., 2016). ​ In the subsequent section, we present various clinical trials focusing on miRNAs, showcasing the therapeutic endeavors in this field.

Research findings suggest that miR-34, a miRNA that acts as a tumor suppressor, is directly controlled by p53 and has the ability to regulate the expression of multiple oncogenes (Liu et al., 2011; Adams et al., 2016a; Adams et al., 2016b). In a phase I study conducted in multiple centers (NCT01829971/NCT02862145), the safety of MRX34, liposomes containing MicroRNA miR-RX34, was assessed in patients with primary liver cancer, metastatic cancer, or hematologic malignancies involving the liver. Although MRX34 displayed some antitumor effects, immune-related toxicity became evident among several participants during the phase Ib examination. The final outcomes of the tests are yet to be published. Another study (NCT02369198) conducted a phase I trial to evaluate the maximum tolerance dose (MDT) of TargomiRs, minicells loaded with miR-16 mimic miRNA and aimed at EGFR, in patients with relapsed malignant pleural mesothelioma and NSCLC (Reid et al., 2013). 26 patients were treated with TargomiRs and it was determined that 5×10^9^ TargomiRs once weekly was the MDT. The proportion of patients who achieved an objective response was one of 22 (5%). Therefore, further investigation into the use of TargomiRs in combination with chemotherapy or immune checkpoint inhibitors is warranted. H19, a frequently upregulated lncRNA in various cancer types, has been extensively utilized by researchers to achieve cancer-specific expression of downstream sequences (Bhan et al., 2017). BC-819, also known as DTA-H19, is a DNA plasmid that encodes diphtheria toxin A (DTA) under the control of the H19 promoter and can be administered as a complex with polyethylenimine (PEI) (Gofrit et al., 2014). Ongoing clinical trials involving BC-819 primarily focus on bladder cancer. A phase II, open-label, single-arm, monotherapy study conducted across multiple centers aims to examine 140 non-muscle-invasive bladder cancer patients who have shown resistance to *Bacillus* Calmette-Guerin (BCG) treatment (NCT03719300).

## 5 Discussion

Initially, cancer was thought to be an inherited disease, but over the past few decades, researchers have established a strong link between epigenetic factors and tumorigenesis. With the help of next-generation sequencing technology, people have successfully discovered changes in epigenome control genes that play a key role in tumor development. For example, single-cell chromatin overall omic-scale landscape sequencing (scCOOL-seq) revealed a new set of candidate biomarkers—ZNF667 and ZNF667-AS1, whose expression is associated with better prognosis in patients with pancreatic ductal adenocarcinoma by influencing the proliferation of cancer cells (Fan et al., 2022). In addition, in the early stages of the disease, people can detect epigenetic modification changes in blood or tissue marker genes for early diagnosis of the disease (Davalos and Esteller, 2023). For example, in 2014, the FDA approved Cologuard for screening for colorectal cancer. It is based on the detection of specific epigenetic changes in fecal DNA.

The epigenetic dysregulation that occurs in cancer makes epigenetic mechanisms a new target for drug development. There are only a few epigenetic drugs approved by the FDA, but more and more epigenetic drugs have been put into the clinical research of cancer treatment, and have achieved remarkable results. Epigenetic modifiers mainly regulate histone post-transcriptional modification and nucleosome assembly. In the future, epigenetic modifiers will also regulate key processes such as DNA repair, genome integrity and RNA splicing. Still, there are problems with epigenetic drugs that need to be addressed. For example, targeted delivery of drugs by siRNA has potential off-target problems. Drugs that interfere with DNA methylation modification, such as azacitidine and decitabine, can cause bone marrow suppression and gastrointestinal symptoms, while epigenetic drugs that can interfere with histone modification are generally cytotoxic. Answers to these questions may better promote the role of epigenetics in treating disease.

In the future, combination therapy based on epigenetic regulators may be the development direction of epigenetic therapy for solid tumors. First, epigenetic regulators can regulate NK cell function, restore depleted T cells and increase immune cell infiltration. Therefore, the combination of epigenetic drugs may enhance the efficacy of tumor immunity. Second, there is some resistance to traditional anticancer drugs. Drug-resistant cancer cells exhibit high chromatin inhibition and elevated levels of KDM5A. Epigenetic drugs can inhibit the development of drug resistance and enhance drug efficacy. In conclusion, the related research and development of epigenetic drugs will become an important way of cancer treatment.
